# Tinnitus and sound intolerance: evidence and experience of a Brazilian group^☆^

**DOI:** 10.1016/j.bjorl.2017.12.002

**Published:** 2017-12-24

**Authors:** Ektor Tsuneo Onishi, Cláudia Couto de Barros Coelho, Jeanne Oiticica, Ricardo Rodrigues Figueiredo, Rita de Cassia Cassou Guimarães, Tanit Ganz Sanchez, Adriana Lima Gürtler, Alessandra Ramos Venosa, André Luiz Lopes Sampaio, Andreia Aparecida Azevedo, Anna Paula Batista de Ávila Pires, Bruno Borges de Carvalho Barros, Carlos Augusto Costa Pires de Oliveira, Clarice Saba, Fernando Kaoru Yonamine, Ítalo Roberto Torres de Medeiros, Letícia Petersen Schmidt Rosito, Marcelo José Abras Rates, Márcia Akemi Kii, Mariana Lopes Fávero, Mônica Alcantara de Oliveira Santos, Osmar Clayton Person, Patrícia Ciminelli, Renata de Almeida Marcondes, Ronaldo Kennedy de Paula Moreira, Sandro de Menezes Santos Torres

**Affiliations:** a Universidade Federal de São Paulo (Unifesp-EPM), Escola Paulista de Medicina, São Paulo, SP, Brazil; b Universidade do Vale do Taquari (Univates), Lajeado, RS, Brazil; c University of Iowa, Iowa, USA; d Universidade de São Paulo (USP), Faculdade de Medicina, São Paulo, SP, Brazil; e Fundação Educacional D. André Arcoverde (FAA), Faculdade de Medicina de Valença, Valença, RJ, Brazil; f Universidade Federal do Paraná (UFPR), Hospital de Clínicas, Centro de Zumbido, Curitiba, PR, Brazil; g Instituto Ganz Sanchez, São Paulo, SP, Brazil; h Hospital Samaritano São Paulo, Clinica Lima Gürtler, São Paulo, SP, Brazil; i Universidade de Brasília (UnB), Brasília, DF, Brazil; j Universidade Federal de Minas Gerais (UFMG), Hospital das Clínicas, Hospital Felício Rocho, Belo Horizonte, MG, Brazil; k Rede Mater Dei de Saúde, Mater Dei Contorno, Belo Horizonte, MG, Brazil; l Irmandade Santa Casa de Misericórdia de São Paulo, Hospital Santa Izabel, São Paulo, SP, Brazil; m Centro de Otorrinolaringologia da Bahia (CEOB), Salvador, BA, Brazil; n Universidade Federal do Rio Grande do Sul (UFRGS), Hospital de Clínicas de Porto Alegre, Porto Alegre, RS, Brazil; o Centro de Tratamento e Pesquisa em Zumbido, Belo Horizonte, MG, Brazil; p Pontifícia Universidade Católica de São Paulo (PUC-SP), Derdic, São Paulo, SP, Brazil; q Faculdade de Ciências Médicas da Santa Casa de São Paulo, São Paulo, SP, Brazil; r Hospital do Servidor Estadual de São Paulo (IAMSPE), São Paulo, SP, Brazil; s Faculdade de Medicina do ABC, Santo André, SP, Brazil; t Universidade Federal do Rio de Janeiro (UFRJ), Rio de Janeiro, RJ, Brazil; u Hospital Federal da Lagoa, Rio de Janeiro, RJ, Brazil; v Faculdade de Medicina de Jundiaí, Jundiaí, SP, Brazil; w Santa Casa de Belo Horizonte, Belo Horizonte, MG, Brazil; x Hospital Otorrinos, Feira de Santana, BA, Brazil

**Keywords:** Tinnitus, Hyperacusis, Hearing loss, Hearing aids, Zumbido, Hiperacusia, Perda auditiva, Auxiliares de audição

## Abstract

**Introduction:**

Tinnitus and sound intolerance are frequent and subjective complaints that may have an impact on a patient's quality of life.

**Objective:**

To present a review of the salient points including concepts, pathophysiology, diagnosis and approach of the patient with tinnitus and sensitivity to sounds.

**Methods:**

Literature review with bibliographic survey in LILACS, SciELO, Pubmed and MEDLINE database. Articles and book chapters on tinnitus and sound sensitivity were selected. The several topics were discussed by a group of Brazilian professionals and the conclusions were described.

**Results:**

The prevalence of tinnitus has increased over the years, often associated with hearing loss, metabolic factors and inadequate diet. Medical evaluation should be performed carefully to guide the request of subsidiary exams. Currently available treatments range from medications to the use of sounds with specific characteristics and meditation techniques, with variable results.

**Conclusion:**

A review on tinnitus and auditory sensitivity was presented, allowing the reader a broad view of the approach to these patients, based on scientific evidence and national experience.

## Resumo

**Introdução:**

Zumbido e intolerância a sons são queixas frequentes e subjetivas que podem ter impacto na qualidade de vida do paciente.

**Objetivo:**

Apresentar uma revisão dos principais pontos incluindo conceitos, fisiopatologia, diagnóstico e abordagem do paciente com zumbido e sensibilidade a sons.

**Método:**

Revisão da literatura com levantamento bibliográfico na base de dados da LILACS, SciELO, Pubmed e MEDLINE. Foram selecionados artigos e capítulos de livros sobre zumbido e sensibilidade a sons. Os diversos tópicos foram discutidos por um grupo de profissionais brasileiros e as conclusões descritas.

**Resultados:**

A prevalência de zumbido tem aumentado ao longo dos anos, muitas vezes associado à perda auditiva, fatores metabólicos e erros alimentares. A avaliação médica deve ser realizada minuciosamente no sentido de orientar a solicitação de exames subsidiários. Os tratamentos disponíveis atualmente variam de medicamentos à utilização de sons com características específicas e técnicas de meditação, com resultados variáveis.

**Conclusão:**

Foi apresentada uma revisão sobre os temas, permitindo ao leitor uma visão ampla da abordagem dos pacientes com zumbido e sensibilidade auditiva baseada em evidências científicas e experiência nacional.

## Introduction

Tinnitus can be defined as a symptom related to the conscious perception of an auditory sensation in the absence of external sound stimuli. It is a prevalent otological symptom, that can have severe physical and emotional consequences.^1^, ^2^, ^3^

Tinnitus is often accompanied by some intolerance to external sounds, which may be:

(a) Hyperacusis: clinically, the patient has light to moderate sensitivity to sounds intensity, with physical discomfort. The Loudness Discomfort Level (LDL) measurement is below 90–100 dB NA. It is the most frequently studied type in studies.
(b) Misophonia: clinically, the patient has aversion to specific sounds, usually low and repetitive, which trigger strong discomfort. It depends on associations of the auditory pathway with the limbic system and on previous negative experience with these sounds, regardless of intensity. Although common in practice, it has been described only recently.
(c) Phonophobia: clinically, the patient is afraid of exposure to the sounds before they reach the discomfort level.
(d) Recruitment: it is a cochlear phenomenon characterized by injury to outer hair cells (OHCs), of which auditory sensation is disproportionate to the increase in the physical intensity of the sound. Audiometry and immitanciometry show that there is a reduction in the auditory dynamic field.^4^, ^5^

## Tinnitus classification

There are several types of classifications,^6^ and the ones most often used are those show in Table 1.

**Table 1:** Tinnitus classification.

Primary (auditory or sensorineural)	Tinnitus may or may not be associated; sensorineural hearing loss (SHL); idiopathic (no other cause is observed except SHL)
Secondary (para-auditory)	Tinnitus associated with a specific cause (other than SHL) or some identifiable organic cause
Acute	Symptom onset less than 6 months before
Chronic	Symptom for 6 months or more
Rhythmic	It can have vascular origin (synchronous with heart beat), muscular, auditory tube and intracranial hypertension
Non-rhythmic	Related to the auditory system
Objective	Perceived by the examiner
Subjective	Perceived only by the patient

## Epidemiology

According to the World Health Organization, 278 million people have tinnitus – approximately 15% of the world's population. This prevalence increases to 35% in individuals over 60 years of age.^1^ In a population study carried out in the city of São Paulo, there was a prevalence of 22%, more common in females (26% in women versus 17% in men) and increasely more common with aging. In most cases, tinnitus is mild and intermittent, and does not prompt the individual to seek medical assistance.^3^, ^7^ Hearing loss is associated with tinnitus in approximately 85–96% of cases, and only 8–10% of affected individuals have normal hearing.^8^

There are few studies on hyperacusis prevalence, but it is estimated to occur between 8–15% in the general population, around 3% in children and in 25–40% of those individuals who have tinnitus.^9^, ^10^

## Pathophysiology of tinnitus and hyperacusis

There are several hypotheses related to the mechanisms of tinnitus generation and hyperacusis. These can be divided didactically into peripheral and central mechanisms.

### Peripheral mechanisms

• Spontaneous Otoacoustic Emissions (OAE): weak acoustic signal generated by the electromechanical activity of the OHCs and captured by microphones in the external acoustic meatus. Controversial mechanism, since individuals without a complaint of tinnitus may have spontaneous OAE.^11^
• Tectorial membrane detachment: cell injury cause by ototoxic agent or acoustic trauma, for instance, affects first the OHCs and, later, the inner hair cells (IHCs).^2^, ^12^ If the lesion affects only the OHCs, there may be loss of the tectorial membrane support and its direct contact with the IHCs, which generates sustained depolarization^13^ that can be perceived by the Central Nervous System (CNS) as tinnitus.^14^
• Disproportionate OHC lesion: the afferent pathway informs the CNS of the OHC position in relation to the tectorial membrane, and the efferent path regulates the length of the OHCs after information processing. As the efferent inhibitory impulse is the result of the summation of afferent impulses, there is a decrease in efferent activity. Since each efferent fiber innervates approximately 100 OHCs, this reduction in inhibition can affect areas of the basilar membrane with normal OHCs, causing them to contract freely; this stimulates the IHCs of these regions which could be responsible for the production of tinnitus.^15^
• Neurotransmitter involvement: glutamate is the main excitatory neurotransmitter inside the cochlea, and an increase in its levels could increase cochlear activity, leading to the onset of tinnitus.^16^ Physical or psychological stress increases dynorphin levels (opioid peptide), which potentiates glutamate action on NMDA (N-methyl-d-aspartate) receptors. The peripheral auditory lesion can lead to neuroplasticity of the auditory cortex, and this central reorganization, mediated by serotonin, may be responsible for the tinnitus.

### Central mechanisms

• Increased spontaneous neural activity in the auditory pathway and in the dorsal cochlear nucleus: the cochlear lesion reduces the afferent stimulus and affects the autoregulation of the central auditory pathway with increased spontaneous activity, interpreted as tinnitus. Autoregulation would lead to exaggerated electrical stimulation in response to sound, resulting in hyperacusis.^2^, ^17^
• Cross-talk between the fibers of the VIII cranial nerve: when loss of the myelin sheath (by tumor compression or vascular loop) leads to the formation of atypical neural connections between nerve fibers, the resulting spontaneous activity can be interpreted by the auditory cortex as tinnitus.^18^
• Neural plasticity and alteration in the Tonotopic Map: the reorganization of the tonotopic map in the cochlear nuclei, in response to a cochlear lesion, would lead to the activation of certain regions of the auditory cortex, which results in the perception of tinnitus and hyperacusis.^19^

### Neurophysiological model

**Figure 1:**
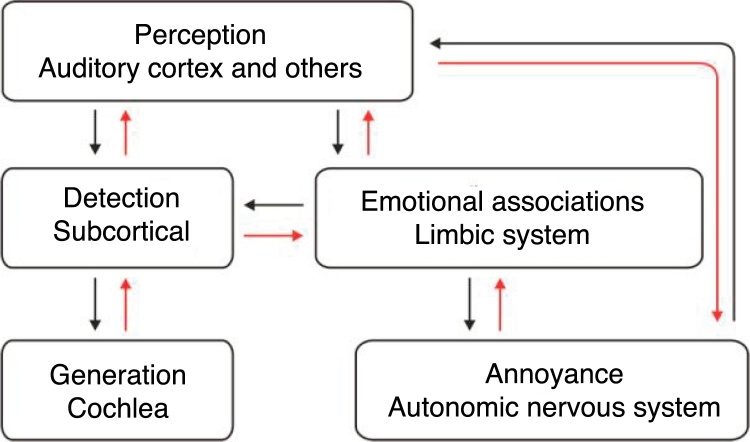
Schematic diagram of the neurophysiological model developed by Pawell Jastreboff.

The neurophysiological model described by Pawell Jastreboff in 1990^2^ explains the process that causes an individual to be disturbed by tinnitus. It can be divided into three phases: generation, detection and perception (Fig. 1). Generation occurs mainly in the peripheral auditory pathways (cochlear or auditory nerve dysfunction). Detection occurs in the subcortical centers and the perception in the auditory cortex.

Depending on the impact of tinnitus on the affected individual, areas of the CNS (limbic system, frontal cortex, autonomic nervous system) and areas of negative association that increase patient discomfort, may be activated.^13^

### Somatosensory Tinnitus

The psychoacoustic characteristics of tinnitus (intensity and frequency) and its location may be altered in some patients, even temporarily, by different stimuli: forced contractions of the head, face and neck muscles, pressure of myofascial trigger points (TP). This characterizes somatosensory tinnitus.^20^ Somatic influences on auditory perception are a fundamental physiological characteristic inherent to every human being, not limited to patients with tinnitus. Temporomandibular joint (TMJ) disorders may affect the auriculotemporal nerve with disinhibition of the dorsal cochlear nucleus activity, through the serotonergic somatosensory pathway.

## Medical evaluation

Tinnitus may be the initial manifestation of several systemic ear diseases or appear during the course of the latter. The diagnosis and treatment of these diseases can lead to tinnitus abolition or amelioration. The patient's clinical history should be directed to the complaint:

(a) Characterization

- Type of sound: it is noteworthy to ask what type of sound the patient hears: waterfall, whistle, cicada, etc. Pulsatile tinnitus can be caused by vascular diseases. In that case, it is important to evaluate any synchronicity with the heart beat and variations with the decubitus position, exercises, stress, etc. The description of sound as fast and repetitive clicks, although without synchronicity with the pulsation, suggests a diagnosis of myoclonus.
- Time of symptom onset: neuroplastic alterations tend to be greater in more chronic tinnitus.
- Laterality and symmetry: unilateral or asymmetrical tinnitus may indicate retrocochlear diseases and should be investigated in a manner similar to that for an unilateral or asymmetric sensorineural hearing loss.
- Continuous or intermittent: the tinnitus may be continuous or intermittent.
- Modulation: represents the immediate change of tinnitus (intensity, frequency or location) in the presence of some stimulus: head movement, position, muscle contraction, stress, noise, etc.

(b) Associated symptoms

- Hearing loss: common in patients with tinnitus, it may indicate an underlying otologic disease.
- Vertigo and dizziness: a specific neurotological diagnosis (dizziness, lightheadedness, decreased visualacuity, fainting sensation, etc.), periodicity (continuous or attacks), auditory symptoms (aural fullness or hypoacusis, worsening of tinnitus, autophony) and other balance complaints (lateropulsion, oscillopsia, gait changes).
- Exposure to noise: it can be occupational or recreational. The exposure period, frequency and intensity should be characterized. It is also important to verify the exposure to impact noises such as fireworks, shots and explosions, or barotrauma.
- Otologic history: it should include all otologic symptoms. In the presence of pain with normal otoscopy, questions should be asked about dental symptoms: bruxism or dental tightening, inappropriate habits such as chewing gum, biting pens or pencil, etc.
- General symptoms and personal history: cardiovascular, metabolic, hormonal, neurological or psychiatric diseases and sleep quality.
- Use of medication and illicit drugs: current use or at the time of the tinnitus onset, paying special attention to the use of ototoxic drugs.

(c) Improvement and worsening factors

Patients with tinnitus usually report worsening in silent environments. Exposure to noise may be the temporary worsening factor for tinnitus; for instance, in the tonic tensor tympani syndrome and in the semicircular canal dehiscence.^21^ Studies on foods that interfere with tinnitus are scarce. Fish consumption one or more times a week and dairy restriction seem to reduce persistent tinnitus.^22^ Hearing loss is an important risk factor, as well as exposure to noise, hyperlipidemia, asthma, osteoarthritis, rheumatoid arthritis and thyroid diseases^23^ and systemic arterial hypertension.^24^

Depression and anxiety may be associated, especially in the most uncomfortable cases.^25^, ^26^ Among the medications implicated in the onset of tinnitus are antibiotics (aminoglycosides), diuretics (furosemide), chemotherapeutics (cisplatin), as well as nonsteroidal anti-inflammatory drugs and quinine.^27^

(d) Diet and habits

- Smoking: it may have an ototoxic effect.^28^
- Alcoholism: it can alter endolymph density, producing transient dysfunction of the outer and IHCs.
- Xanthine consumption: the three main xanthine alkaloids are caffeine (coffee), theophylline (teas) and theobromine (cocoa), substances present in cola drinks, analgesic drugs, antihistamines, etc. Although excessive consumption of xanthines (above 250 mg/day or 3 coffees/day) is considered an exacerbating factor for tinnitus and dizziness, this is controversial.^29^
- Consumption of fast-absorbing sugars and carbohydrates: it may cause tinnitus or worsen existing tinnitus by hyperinsulinism and alterations in the endocochlear potential.^30^, ^31^
- Prolonged fasting: The Na/K pump mechanism that is responsible for the endocochlear potential is energy dependent and the inner ear does not store energy. Prolonged fasting (over 3 h) is related to energy deficit and alteration in the endocochlear potential, which may worsen tinnitus.^31^

### Physical examination

It should include otoscopy, oroscopy, anterior and posterior rhinoscopy. The otoscopy of patients with vascular tinnitus may show a reddish retrotympanic area (paraganglioma, aberrant internal carotid artery in the middle ear, high and dehiscent jugular bulb, among others). Periauricular, periorbital, cervical auscultation and neck palpation can provide signs of vascular malformations, arteriovenous fistulas, or venous “hum” – a situation in which tinnitus increases with contralateral cervical rotation and decreases with ipsilateral rotation. In the case of tinnitus related to the patent auditory tube, synchrony with respiration is observed and otoscopy reveals motion of the tympanic membrane during inspiration and expiration.

Pulsatile tinnitus may be associated with benign intracranial hypertension. The presence of papilledema in the fundoscopic examination of the eye assists in the diagnosis.

The evaluation of cranial nerves identifies central etiologies, especially in patients with headache, paresthesia, diplopia or dizziness. The study of cranial pairs, cerebellar tests and the assessment of upper and lower limb muscle strength should be included. Tumors of cranial nerves IX, X, XI may generate pulsatile tinnitus by altering blood flow through the jugular bulb inside the jugular foramen.^32^

#### TMJ evaluation

When associated with tinnitus, TMJ disorders may also exhibit otalgia, hearing loss, ear fullness, hyperacusis, and dizziness. Physical examination should be performed with special attention to pain in the TMJ region (spontaneous, with chewing and during palpation); crackling of the joint; difficulty in maximal mouth opening. Forced jaw protrusion or lateralization can modulate the tinnitus.

#### Nasofibroscopy

Some types of myoclonus can cause tinnitus via contraction of the middle ear muscles (tensor tympani and stapedius muscles)^33^ or muscles of the palatal region (soft palate and pharyngeal).^34^ Palatal myoclonus may be inhibited by opening the mouth; thus, palatal observation through nasofibroscopy is essential.^32^ It is possible to observe rhythmic movements in the soft palate region.

#### Questionnaires

It is critical to distinguish between tinnitus and tinnitus reactions. For that purpose, questionnaires assessing and quantifying tinnitus and its effects on the patient's life can be used.

The most often used are: Tinnitus Questionnaire,^35^ Tinnitus Handicap Inventory (THI),^36^ Tinnitus Reaction Questionnaire^37^ and Tinnitus Handicap Questionnaire.^38^ All show good reproducibility and internal consistency and, therefore, the choice of the tool should be made based on the existence of the adapted version in the country's language and familiarity with the questionnaire. The THI, developed by Newman,^36^ has been validated in several languages, including Portuguese.^39^, ^40^ The tool consists of 25 questions with a score varying from 0 to 100; the higher the score, the greater the effect of tinnitus on the patient's life. The Tinnitus Questionnaire is a long questionnaire consisting of 52 questions and has a shorter version, called the “Mini Tinnitus Questionnaire” (or Mini-TQ).^41^ The Mini-TQ has a Portuguese version, with 12 questions that mainly evaluate the annoyance with tinnitus and how it affects the individual's daily life.^42^

The Visual Analog Scale (VAS) is used to measure subjective phenomena, and is used widely in pain assessment. It provides a simple and quick measurement of intensity and degree of annoyance, using numbers from 0 to 10.^43^

## Complementary assessment

### Hearing tests

Hearing tests help to diagnose hearing loss, as well as to aid in the diagnosis leading to specific treatment. A second reason to perform these tests is to establish if the patient is a candidate for hearing aid use.

#### Audiometry

Speech and pure-tone audiometry, and immittance testing: assess auditory acuity, type and degree of hearing loss, thus directing the medical evaluation.

- High-frequency audiometry: it evaluates the frequencies of 9000–20,000 Hz, corresponding to the base of the cochlear. There is still no consensus for auditory thresholds at these frequencies.
- Acuphenometry: tinnitus can be measured to demonstrate to patients that their tinnitus is real, help in patient counseling, and assist in sound therapy prognosis.
- Tinnitus Matching (TM) attempts to establish the pitch (frequency) and loudness (intensity) of tinnitus.
- Minimum Masking Level (MML) evaluates the lowest sound intensity that masks the tinnitus.
- Residual Inhibition or Suppression Effect: evaluates the temporary inhibition of tinnitus, after stimulation with broadband noise 10 dB above MML, for 60 s. When present, partial or total inhibition occurs after the end of the stimulus and lasts a brief time until the tinnitus returns to its previous level.
- LDL: assesses the noise discomfort threshold. Pure or pulsating tones are presented, with a gradual increase of 5 in 5 dB, between 500 and 8000 Hz, with 1-second inter-stimulus intervals and 1-second duration. The patient should raise his/her hand when the sound is at such intensity that he or she does not want to hear it (initial discomfort), in order to evaluate the lowest sound intensity that causes discomfort.

### Electrophysiological and electroacoustic tests

Electrophysiological and electroacoustic tests help to manage the patient with a tinnitus complaint in two ways: by facilitating the investigation of the causal factor and the understanding of the pathophysiological mechanisms involved.

(1) Tests requested for topographic assessment of tinnitus:

(a) Patient with tinnitus and/or auditory hypersensitivity and sensorineural hearing loss:

- Brainstem Auditory Evoked Potential (BAEP): when the objective is to evaluate the auditory nerve and/or brainstem (tumors, demyelination or dyssynchrony).
- Transient/Distortion-Product OAEs: Normal OAEs and altered audiometric thresholds suggest a noncochlear cause. In this situation, imaging tests and/or BAEP assessment should be requested.^44^

(b) Patient with tinnitus and/or auditory hypersensitivity without hearing loss:

- Transients/Distortion-Product OAE: its alteration suggests that such a cochlear lesion was not sufficient to generate effects on tonal audiometry, similar to a subclinical hearing loss.
- BAEP: changes in the auditory nerve and/or brainstem can also occur without associated hearing loss.

(c) Patient with tinnitus and/or auditory hypersensitivity and dizziness regardless of hearing loss:

- Electrocochleography: it is indicated to investigate endolymphatic hydrops.
- VEMP or Vestibular Evoked Myogenic Potential: it assesses the involvement of the otolithic organs and their nerve pathways. It evaluates the involvement of otolithic organs and the associated pathways.

(2) Examinations requested for scientific research and understanding of pathophysiological mechanisms of tinnitus and/or sound hypersensitivity:

- BAEP: The increased amplitude ratio of III/I and V/I waves suggests greater electrical activity in the ventral cochlear nucleus and inferior colliculus, while the increase in the latency of the V wave and the III-V interpeak suggest changes in electrical conduction, whether primary or secondary to the tinnitus.^45^
- P300: altered latencies may suggest changes in functions such as attention and short-term auditory memory.^46^

Contralateral suppression of OAE: it evaluates the involvement of the corticofugal auditory efferent system in the origin and maintenance of tinnitus and auditory hypersensitivity.^47^

### Laboratory tests

Some tests may provide relevant information to the investigation of etiological, predisposing, or coadjuvant factors in patients with tinnitus.

- *Fasting glucose and glycated hemoglobin*: may be replaced or supplemented by the 3-h glucose tolerance test and insulin curve. In this case, the objective is to evaluate glucose metabolism and the consequent insulin production, with hyperinsulinemia and reactive hypoglycemia being possible suspicions, or also, disaccharidase alterations (lactose intolerance).^31^
- *Total cholesterol and fractions and Triglycerides*: allow investigating factors that cause blood hyperviscosity or atherosclerotic plaques.^48^
- Free T4, TSH, anti-peroxidase antibodies and antithyroglobulin antibodies: search for early thyroid abnormalities.^23^, ^49^

*Zinc*: especially in elderly patients, or in the postoperative period of bariatric surgery, in which this trace element may be deficient.^50^

Other tests may be requested, such as the whole blood count; Magnesium^51^; Vitamin B12^52^; Folic acid^53^; Cortisol^54^; Serotonin^54^; Vitamin D; Ferritin.

### Laboratory tests in patients with hyperacusis

To date, laboratory abnormalities related to hyperacusis and misophonia have not been identified.

### Imaging diagnosis

(1) Imaging tests in non-rhythmic tinnitus

- Cervical X-ray: allows the diagnosis of cervical diseases such as compression of vessels by the alar process of the vertebrae.
- Temporal bone tomography: this test allows the identification of middle and inner ear diseases that may be associated with non-rhythmic tinnitus.^30^, ^55^
- Magnetic resonance imaging of the inner ear with gadolinium: it helps in the identification of neurologic diseases and is essential to rule out retrocochlear disease in patients with unilateral or asymmetrical tinnitus, with or without hearing loss.^30^, ^55^

(2) Imaging tests in patients with rhythmic tinnitus

- Temporal bone CT with contrast: it may clarify possible retrotympanic pulsatile lesions identified at otoscopy: aberrant carotid artery, high jugular bulb and glomus tumor.^56^
- Doppler ultrasound of carotid, vertebral and subclavian arteries: it investigates atherosclerotic disease that can change laminar blood flow. These patients usually have hypercholesterolemia, hypertension, diabetes mellitus, angina and smoking habit.^56^
- Transcranial Doppler ultrasound: it evaluates atherosclerosis in the intracranial carotid system, in addition to arterial malformations and other vascular abnormalities.^56^
- Angiotomography: it is considered the gold standard in pulsatile tinnitus. It shows the association of the vessels with the bony structures of the temporal bone.^57^
- Magnetic resonance angiography: the arterial phase is important in the diagnosis of carotid artery dissection. The venous phase allows the diagnosis of fistulas and arteriovenous malformations, but with less sensitivity than angiography. It helps to investigate pulsatile tinnitus, palatal or stapedius myoclonus, as there may be associated neurological lesions. It also helps in the diagnosis of benign intracranial hypertension, which usually affects middle-aged, Caucasian, obese women with complaints of headache, visual blurring, double vision and/or pain on ocular movement.^58^
- Angiography: it allows the diagnosis of small arteriovenous fistulas, but it should be the last option due to its risks. It also becomes important in patients with glomus tumor to assess the vascular supply and the possibility for embolization.^59^

**Figure 2:**
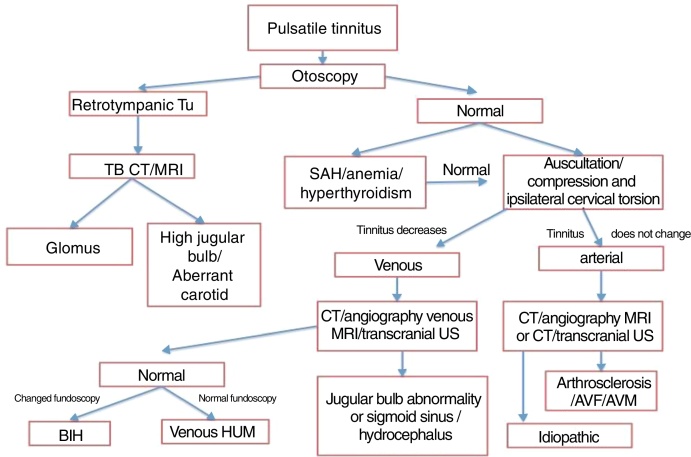
Flowchart of pulsatile tinnitus investigation. Tu, tumor; CT, computed tomography; TB, temporal bones; SAH; systemic arterial hypertension; MRI; magnetic resonance imaging; US, ultrasound; BIH, benign intracranial hypertension; AVF, arteriovenous fistula; AVM, arteriovenous malformation.

Fig. 2 shows a suggested investigation flowchart for pulsatile tinnitus.

## Treatment and rehabilitation

Counseling is a principal component of two important therapies for hyperacusis. In Hyperacusis Activities Treatment, guidelines on thinking, emotions, hearing, concentration and sleep are proposed, in addition to the acoustic therapy that will be discussed below. This counseling is directed by a specific questionnaire to recognize the areas most affected.^60^ In Tinnitus Retraining Therapy (TRT), the counseling is also directed toward auditory hypersensitivity – hyperacusis and misophonia. In the latter, results exceed 76% for isolated hyperacusis or 83% for isolated misophonia.^61^

Ear protective devices can increase a person's hearing sensitivity and we often see patients with hyperacusis wearing them. They should be encouraged to avoid such protection because it reinforces an association between auditory signals and distress and potentiates existing fear and underlying anxiety.^62^

### Pharmacological and surgical treatment of hyperacusis

Successful treatments have already been described in patients with hyperacusis using alprazolam,^63^ carbamazepine,^21^ fluoxetine and fluvoxamine^64^ and citalopram.^17^

Regarding the surgical treatment of hypersensitivity, isolated reports in the literature range from fascia reinforcement placed on the oval and round windows, obliteration of semicircular canal dehiscence and even labyrinthectomy.^65^, ^66^

### Pharmacological treatment of tinnitus

Although to date there is no drug approved by the F.D.A. (Food and Drug Administration) with a specific indication for tinnitus treatment, there is no reason to believe that tinnitus cannot be pharmacologically treated.

There is no consensus regarding the duration of treatment, as it should be individualized. In most studies, the treatment is carried out for 2–3 months.

Didactically, one can classify drugs for tinnitus treatment in three large groups (Table 2):

- Drugs that improve vascular supply and inner ear metabolism;
- Drugs that act on ion channels;
- Drugs that act on neurotransmitters.

**Table 2:** Classification of medications for tinnitus treatment.

Mechanism of action	Medications
Improves vascular supply and internal ear metabolism	Trimetazidine
	Ginkgo biloba extract
	Vinpocetine
	Betahistine

Effect on ion channels	Carbamazepine
	Gabapentin
	Nimodipine

Effect on Neurotransmitters	Caroverine
	Memantine
	Acamprosate
	Clonazepam
	Baclofen
	Sertraline
	Trazodone
	Cyclobenzaprine
	Pramipexole
	Sulpiride

## Vascular and metabolic supply of the inner ear

The indication for the use of trimetazidine, which presumably acts on cochlear metabolism, has recently been withdrawn for treatment of dizziness and tinnitus.

Ginkgo Biloba shows diverse results in systematic reviews. A recent study assessing 1543 patients, concluded that there is limited evidence to demonstrate its efficacy in tinnitus treatment.^67^

A single study analyzed the efficacy of vinpocetine demonstrating some positive results in tinnitus associated with acoustic trauma.^68^

Betahistine may be effective when tinnitus is associated with dizziness.^69^

## Ion channels

There have been studies on drugs that act on the sodium, potassium and calcium channels.

### Sodium channels

The prototype of this approach was the verification of the immediate effects of intravenous lidocaine on tinnitus.^70^ However, because of the administration route and possible side effects, there is no clinical application for lidocaine in tinnitus treatment. A systematic review of the use of anticonvulsants (including carbamazepine and lamotrigine) concluded that there is no evidence that anticonvulsants have a significant positive effect on tinnitus treatment.^71^

### Potassium channels

There is only experimental evidence of the effect of this type of drug on tinnitus, including the *flindokalner*.^72^

### Calcium channels

Although the mechanism of action for gabapentin is not fully understood, it is believed that calcium channel blocking is the main mechanism. A recent systematic review concluded that there is not enough clinical evidence to recommend the use of gabapentin for the treatment of tinnitus.^73^

A single open-label study evaluated the effects of nimodipine with a poor outcome.^74^

## Neurotransmitters

### Glutamate

This is the main excitatory neurotransmitter and the effects of excitotoxicity have been well experimentally documented.^16^ Caroverine blocks AMPA and NMDA receptors, but the promising initial results were not replicated.^75^ It is not commercially available in Brazil. Memantine is an NMDA blocker that has shown promising results in experimental studies,^76^ but not in a clinical trial.^77^ Acamprosate is an NMDA blocker that also has GABAergic activity. The results were positive in two studies,^78^, ^79^ but the drug is not available in Brazil. The clinical trial of the NMDA blocker esketamine (AM-101) for the treatment of tinnitus for up to 3 months in duration, using intratympanic injections, is currently in phase 3, with very promising phase 2 results.^80^

### GABA

A recent systematic review of the benzodiazepines used in the treatment of tinnitus has concluded that there is evidence, albeit not robust, of positive effects of clonazepam, a GABA-A receptor agonist, but not of alprazolam or diazepam.^81^ In a randomized cross-over study against Ginkgo biloba, the efficacy of clonazepam in tinnitus relief was demonstrated.^82^ The risk of dependence and side effects (drowsiness, urinary retention, increased eye pressure) require caution for its use.

Experimental evidence for efficacy of the GABA-B agonist baclofen a has not been replicated in clinical trials.^83^

### Serotonin

Selective serotonin reuptake inhibitors (SSRIs) are widely used as antidepressants in the associations between tinnitus, anxiety, and depression. A recent systematic review concluded that there is insufficient evidence of a direct effect of antidepressants on tinnitus.^84^ However, they may be helpful in relieving depression and anxiety associated with it. It is worth adding that there are reports about the onset or worsening of tinnitus with the use of antidepressants, especially the tricyclic ones. A single clinical trial demonstrated the benefits of sertraline, an SSRI, in tinnitus relief.^85^ Trazodone, a serotonin modulator, on the other hand, showed no beneficial effects.^86^

Cyclobenzaprine has several mechanisms of action, including 5 HT-2 receptor antagonism. Clinical studies showed a positive effect in some patients at the dose of 30 mg a day.^87^

### Dopamine

Agonists (piribedil^88^ and pramipexole^89^) and antagonists (sulpiride^90^) have shown beneficial effects in clinical trials, but need confirmation by randomized trials with larger samples. Pyribedil was commercially discontinued in Brazil approximately 2 years ago.

## Other mechanisms of action

Several drugs have already been assessed in clinical studies without meaningful results, such as melatonin, furosemide, atorvastatin, misoprostol, vardenafil. The drugs described for use in tinnitus cases of muscular origin include clonazepam, thiocolchicoside and sumatriptan.^91^ There are clinical reports on the relief of tinnitus of vascular origin with propranolol.^92^

## Use of supplements in the treatment of tinnitus

Dietary supplements may contain vitamins, minerals, herbs or nutritional substances. Considered as “natural” products, they are inexpensive and can be sold without a prescription, but that does not necessarily mean they are safe and effective.^93^ The most often used in the treatment of tinnitus are:

- Vitamin B12: Vitamin B12 deficiency may cause tinnitus and cyanocobalamin replacement may improve the symptom.^52^
- Melatonin (N-acetyl-5-methoxitriptamine): is a hormone secreted by the pineal gland. It acts on sleep control, has neuromodulatory action and antioxidant properties.^94^ The use of melatonin improves sleep, particularly in elderly patients with insomnia. It is inexpensive and safe, and has few adverse effects. Preliminary studies suggest that melatonin has a positive effect on sleep disturbances caused by tinnitus.^95^
- Zinc: this element plays an essential role in function of the cochlear and auditory pathways. Zinc replacement therapy may benefit patients with tinnitus,^96^ especially in subjects with zinc deficiency.

## Sound therapy

### Personal Sound Amplifier (PSA) and sound generator

There is an association between tinnitus and hearing loss in approximately 85–96% of cases.^2^ The decrease in the sound entry into the cochlea results in a decrease in afferent activity to the auditory nerve and the auditory pathways, resulting in changes in all pathways that are responsible for the appearance of tinnitus.^97^ Several studies have shown a reduction of tinnitus annoyance with the use of a hearing aid and/or sound generator.^98^, ^99^ In the American Academy of Otorhinolaryngology guidelines, the use of hearing aids is recommended for patients with hearing loss and tinnitus discomfort, although prospective studies are of low quality and limited by methodological problems (bias, small sample, short time of treatment and with associated treatments, such as sound therapy and counseling). The literature contains some studies that showed improvement in tinnitus discomfort with the use of the hearing aid after one to three months of treatment.^99^, ^100^ Therefore, we suggest that a test with PSA, with or without a sound generator, should be performed for a period equal to or greater than 30 days. The tinnitus improvement occurs in approximately 82% of patients who used an open-fit BTE (Behind-the-Ear) PSA with relief ventilation, and there was no difference between the two groups. However, the preference for open-fit occurs in 66% of the patients.^100^ In clinical practice, we observed that patients who receive both a hearing aid and a sound generator show tinnitus improvement during the prosthesis adaptation period and often choose the device without a sound generator, since the hearing aid helps to mask external noise. Sound amplification improves patient quality of life by favoring hearing and masking tinnitus. Advice and/or guidance regarding tinnitus is essential during the adaptation to hearing aids, aiming to inform the patient about the reason for choosing this treatment to improve both hearing and tinnitus.

#### Sound generator

It can be used in several ways: mixing point – TRT, total and partial sound masking. Or, the lowest intensity capable of promoting tinnitus relief – TAT (Tinnitus Activities Treatment).^101^ Any therapeutic approach applying sound therapy for tinnitus is improved when it is associated with counseling/guidance.

In patients with reduced sound tolerance, it is essential to avoid sound deprivation with the use of an ear protector, which can increase central auditory gain and exacerbate the symptoms of hyperacusis.^4^ In these cases, it is advisable to carry out sound desensitization with the use of a sound generator and hearing aid, gradually introducing the sound for the time that is tolerated by the patient. The adaptation must be slow and progressive, depending on the patient's tolerance. Initially, it is recommended to use the hearing aid in quiet environments and, subsequently, in noisy environments. In severe cases of reduced sound tolerance, it may be necessary to initially apply the sound generator to improve sound tolerance and a later adaptation with a hearing aid.^102^ Currently, the use of digital technology devices has facilitated the programming of these treatments combined with a hearing aid and a sound generator. The use of hearing aids may be valid in patients suffering from tinnitus, even with mild hearing loss.

### Music therapy in tinnitus treatment

#### Customized sounds for tinnitus pitch

Customized acoustic stimuli are adapted according to the patient's hearing and tinnitus pitch. Two types of sounds are offered: relaxing soft music of variable amplitude and a broadband noise similar to white noise. At different phases, the white noise is added or may be removed to help mask the tinnitus. Between 80 and 90% of patients experienced a significant reduction in tinnitus, even when they were not using the device.^103^

#### Fractal tones

Based on the fractal analysis of sounds, five patterns of semi-random tones, similar to bell tunes, were created along with broadband white noise. The proposal is the presentation of melodic tones with a slower time (60–70 beats per minute, similar to resting heart rate), less repetition and without emotional content, which promotes relaxation. A study on the effectiveness of music therapy with fractal tones showed that it does not depend on the nature of the hearing loss or tinnitus characteristics.^104^

#### S-Tones with modulated amplitude and frequency

S-Tones, with modulated frequency and amplitude, produce a robust and synchronized neuronal activity in the auditory cortex. Very slow sounds produce explosions of neural activity, and very fast sounds do not show synchronization, but if presented within a specific interval, the neurons synchronously fire in response to the sound stimulus. Suppression is a physiological process where sounds modulate the activity of the auditory cortex and interrupt tinnitus generation.^105^

#### Spectral notched music

The notched music, tailor-made with the removal of sounds that have the same frequency as the tinnitus, can reduce its volume. The results indicate that the short-term, intensive Tailor-Made Notched Sound Therapy seems to be effective in patients with tinnitus frequencies ≤8 kHz due to the ability of the notched sound to reduce the excitability of hyperactive auditory neurons, which would occur as a result of the strengthening of inhibitory networks, previously weakened in the critical frequency band of tinnitus.

#### Tones for central neural auditory desynchronization

Acoustic Coordinated Reset Neuromodulation aims to reduce abnormal levels of synchronous neural activity in the cerebral auditory cortex, a condition in which a large population of neurons repeatedly and spontaneously fires impulses at the same time. The Neuromodulator CR emits a sequence of low intensity tones, obtained through a mathematical algorithm in which the used tones coincide with the frequency bands adjacent to the tinnitus frequency, individually adjusted for each person. The objective is to stop the increase of the abnormal synchronic firing in brain auditory neurons, responsible for the tinnitus perception.^106^

### Habituation therapy or TRT

TRT or Habituation Therapy aims to change the most activated neural networks in patients with tinnitus annoyance, which are limbic system (hippocampal segment) and the autonomic nervous system, regardless of the source of the tinnitus.^2^ The TRT is based on three pillars:

- Demystification: includes all measures used to reduce or eliminate the negative connotation and activation of the limbic and autonomic nervous systems.
- Counseling: covers all anti-tinnitus measures. The removal of negative associations related to tinnitus, through counseling sessions in which the patient understands the hearing function and the mechanisms of tinnitus perception, may be sufficient to promote the habituation of the reaction, that is, the patient can still perceive the tinnitus, but ceases to be bothered by it.
- Habituation: physiological process characterized by the progressive decline of responses to the same stimulus. The concomitant use of sound therapy may be necessary, for it promotes the constant input of sounds, either through sound generators, hearing aid amplification prostheses or environmental sounds. Habituation occurs if the stimulus is neutral, that is, free of associations and/or connotations with negative emotional states. Patients with hyperacusis, associated or not with tinnitus, are also candidates for treatment with TRT. Table 3 summarizes the proposals and modalities of TRT treatment according to patient groups. The efficacy of habituation therapy is around 84–86%, and may vary according to patient adherence to treatment.^107^, ^108^

**Table 3:** Categorization of tinnitus patients for TRT.

Category	Tinnitus	Hypoacusis	Hyperacusis	Exacerbation with sound	Therapy
0	Low impact	Absent	Absent	Absent	Counseling
1	High impact	Absent	Absent	Absent	Sound generator at the mixing point
2	High impact	Present	Absent	Absent	PSA + Sound enrichment
3	High impact	Absent	Present	Absent	Sound generator close to auditory threshold
4	High impact	Absent	Present	Present	Sound generator close to auditory threshold

### Transcranial magnetic stimulation (TMS)

It is a noninvasive technique of neurostimulation and cortical neuromodulation. The procedure generates repetitive pulses of short duration (100–300 microseconds) and high power (1.5–2.0 Tesla) magnetic field.^109^ Modern TMS systems apply a rapidly changing magnetic field over a specific neural region, inducing electrical activity in the target cortical region. This is typically characterized by disruption of the stimulated target area activity and potential for change in the function of the interrupted area. Therefore, there is neuroplasticity modulation in cortical and thalamic-cortical areas in the same way. TMS is a safe and effective procedure for tinnitus control, but it requires studies with a longer follow-up period.^110^

### Cognitive-behavioral therapy (CBT)

Therapeutic approaches typically involve relaxation training to reduce alertness, creating methods to ignore the tinnitus-related information. CBT aims to identify and change the emotional significance of tinnitus. According to McKenna et al.,^111^ regardless of the symptom's initial cause, the cognitive behavioral process contributes to its severity through intrusive negative thoughts, selective attention, hypervigilance, misconceptions, counterproductive behaviors and a distorted perception of tinnitus. CBT is structured, for a limited time, as an objective to help the patient face certain difficulties, constructing positive thoughts. A 15-year follow-up study demonstrated stability in the improvement,^112^ constituting a good therapeutic option for patients with tinnitus, alone or associated with other types of treatment.

### Acupuncture

Acupuncture has been used in the treatment of tinnitus, similar to the treatment of painful conditions. The needle stimulation causes an electrical discharge that triggers action potentials and influences the activity of the olivocochlear nucleus or the modulation of ascending auditory pathway connections with the limbic system and the amygdala.^6^ In the studies that showed positive results of acupuncture on tinnitus, the time and degree of improvement were very variable.^113^, ^114^, ^115^ Acupuncture is a safe treatment option with no adverse effects, but more studies are required to evaluate its effect on tinnitus.

### Mindfulness

Mindfulness has its origin in Eastern meditative practices. It is defined as a specific form of concentration at the present time, intentionally and without judgment. The practices encompass several techniques, such as breathing exercises, experiencing everyday situations in a conscious way and attention to the sense organs. Studies have shown benefits over tinnitus, reducing annoyance, improving depressive and anxious states and facilitating the acceptance of tinnitus by the patient in up to 87.5%.^116^

### Physical therapy in the treatment of chronic tinnitus

There are anatomical and functional associations between the ears and the mandible, face, nape and neck. Somatosensory tinnitus may be divided according to the topography of symptoms as (1) Craniofacial Dysfunction (CFD), and (2) Craniocervical Dysfunction (CCD).

## CFD

They represent TMJ, masticatory system muscles and/or neuromuscular attachment dysfunctions. In 2003, Tuz et al. evaluated the prevalence of 4 otologic symptoms (otalgia, tinnitus, vertigo and hearing loss) in 200 patients with temporomandibular dysfunction and compared them with an asymptomatic control group.^117^ Tinnitus is at least 2 times more prevalent in patients with CFD. The following are associated factors: dental malocclusion associated with stress and muscular tension, inadequate masticatory power and/or muscular overload during the masticatory process, bruxism, anxiety disorders, systemic diseases that alter bone structures, postural disorders, sleep deprivation. The otologic symptomatology is due to the fact that the ears and the TMJ and its attachments share common innervations (mainly the V and VII cranial nerves) and have neural networks crossed at the level of the brainstem that are able to modulate each other. The cranio-cervical-facial system (mouth, face, head and neck) can modulate the onset and/or perception of tinnitus twice as frequently in tinnitus patients than in the ones without this symptom.^118^ The long-term effects of TMJ treatment have been described in 73 tinnitus patients and in 50 patients with tinnitus and TMJ dysfunction.^119^ In the treatment group, 43% of the patients had tinnitus improvement, 39% had unaltered tinnitus, and 18% had symptom worsening.

## CCD

A recent study showed that among treated patients with non-pulsatile tinnitus, 43% were diagnosed with somatosensory tinnitus.^120^ CCD may be related to inadequate posture of the body. The patient has pain and physiological movement limitation, neck stiffness, sensitivity and/or pain at cervical muscle palpation, radiated pains, headaches and joint dysfunction. Cervical proprioception dysfunction may cause tinnitus and other neurotological symptoms. The patient should be referred early for multidisciplinary evaluation, including otorhinolaryngologist, and physical therapist, physiatrist or orthopedist. In 2012, researchers recruited 71 patients with tinnitus and myofascial pain, divided into one group for deactivation of TP and one placebo group.^121^ There was effective improvement in tinnitus in the active therapy group compared to the placebo. There was an association between pain and tinnitus improvement. The factors correlated with better response to TP deactivation therapy were: (1) the presence of myofascial pain around the ear, (2) the laterality between the symptoms (ipsilateral tinnitus and pain), (3) the reduction of tinnitus by muscle palpation in the initial evaluation.

### Invasive neuromodulation (cochlear implant (CI))

Mertens et al. evaluated the effect of CI on disabling tinnitus of unilateral hearing loss patients for more than 10 years.^122^ Among patients with unilateral anacusis, 83% had tinnitus suppression. Among those with asymmetric hearing loss, 55% reported that the main benefit was auditory improvement. The improvement occurred within the first 3 months after activation. A randomized controlled trial evaluated the effect of CI, unilateral or bilateral, on tinnitus perception in patients with severe bilateral post-lingual deafness.^123^ Before the CI, the prevalence of tinnitus was 42.1%. One year after the CI, the TQ and THI questionnaire scores showed a reduction of 71.4% and 80%, respectively. Although the CI is effective in reducing tinnitus, the patient should be aware of the possibility of its onset or worsening after the procedure. A systematic review assessed the effect of CI on tinnitus in patients with bilateral SHL (sensorineural hearing loss). There was partial improvement of tinnitus in 25 to 72% and total suppression between 8 and 45%. Tinnitus remained unchanged in 0 to 36% of cases and worsened in 0–25% of the time.^124^ A retrospective study by Kloostra et al.^125^ showed that 51.3% of 212 implanted patients had tinnitus. After the CI, 55.6% of the patients reported tinnitus improvement or suppression, while 8.2% had symptom worsening. Among the patients without tinnitus in the preoperative period, 19.6% started to perceive the symptom in the postoperative period.

## Conclusion

It is undeniable that the great medical advances in the last decades have made possible the understanding of phenomena that were previously little known and even neglected, such as tinnitus. The large volume-of information presented in this article is proof of this fact. The complexity and variety of factors that can influence the sensation and degree of discomfort make each patient unique and worthy of individualized attention and care, often with interdisciplinary cooperation. The authors hope that this review may provide support to professionals in assisting them in the understanding, approach and treatment of patients with tinnitus and sound intolerance.

## Conflicts of interest

The authors declare no conflicts of interest.
